# Pre-Analytical Evaluation of Streck Cell-Free DNA Blood Collection Tubes for Liquid Profiling in Oncology

**DOI:** 10.3390/diagnostics13071288

**Published:** 2023-03-29

**Authors:** Inga Medina Diaz, Annette Nocon, Stefanie A. E. Held, Makbule Kobilay, Dirk Skowasch, Abel J. Bronkhorst, Vida Ungerer, Johannes Fredebohm, Frank Diehl, Stefan Holdenrieder, Frank Holtrup

**Affiliations:** 1 Research and Development, Sysmex Inostics GmbH, 20251 Hamburg, Germany; 2 Department of Hematology and Oncology, University Hospital, 53127 Bonn, Germany; 3 Institute of Clinical Chemistry and Clinical Pharmacology, University Hospital, 53127 Bonn, Germany; 4 Department of Pneumology, University Hospital, 53127 Bonn, Germany; 5 German Heart Centre Munich, Institute for Laboratory Medicine, Technical University, 80636 Munich, Germany

**Keywords:** cell-free DNA (cfDNA), circulating tumor DNA (ctDNA), liquid profiling, pre-analytics, precision medicine

## Abstract

Excellent pre-analytical stability is an essential precondition for reliable molecular profiling of circulating tumor DNA (ctDNA) in oncological diagnostics. Therefore, in vitro degradation of ctDNA and the additional release of contaminating genomic DNA from lysed blood cells must be prevented. Streck Cell-Free DNA blood collection tubes (cfDNA BCTs) have proposed advantages over standard K_2_EDTA tubes, but mainly have been tested in healthy individuals. Blood was collected from cancer patients (*n* = 53) suffering from colorectal (*n* = 21), pancreatic (*n* = 11), and non-small-cell lung cancer (*n* = 21) using cfDNA BCT tubes and K_2_EDTA tubes that were processed immediately or after 3 days (BCTs) or 6 hours (K_2_EDTA) at room temperature. The cfDNA isolated from these samples was characterized in terms of yield using LINE-1 qPCR; the level of gDNA contamination; and the mutation status of KRAS, NRAS, and EGFR genes using BEAMing ddPCR. CfDNA yield and gDNA levels were comparable in both tube types and were not affected by prolonged storage of blood samples for at least 3 days in cfDNA BCTs or 6 hours in K_2_EDTA tubes. In addition, biospecimens collected in K_2_EDTA tubes and cfDNA BCTs stored for up to 3 days demonstrated highly comparable levels of mutational load across all respective cancer patient cohorts and a wide range of concentrations. Our data support the applicability of clinical oncology specimens collected and stored in cfDNA BCTs for up to 3 days for reliable cfDNA and mutation analyses.

## 1. Introduction

Continuous improvement of sensitive technologies for the detection and analysis of aberrations in circulating tumor DNA (ctDNA) has paved the way for clinical liquid biopsy applications [1,2]. Because ctDNA is only a marginal amount among a majority of wild-type circulating cell-free DNA (cfDNA), these applications require highly sensitive methods such as barcoded next-generation sequencing or digital PCR approaches [3,4]. Whereas the focus lies on technology development to detect very low mutant allele frequencies (MAFs) as one mutant copy per 10,000 wild-type DNA copies, the influence of pre-analytical steps on the sensitivity of detecting cfDNA remains a major issue [5,6,7,8]. However, this should be given more attention because it can directly affect the sensitivity of ctDNA detection because the release of wild-type DNA due to leukocyte lysis results in the dilution of ctDNA. Pre-analytical steps that can influence sample quality are the blood draw procedure itself (needle diameters and tourniquet), blood collection (tube type, fill volume, and mixing), shipment and storage of the biospecimens, as well as the plasma processing and DNA extraction. However, the most critical step is the storage time between blood sampling and processing. With standard EDTA blood collection tubes, the processing of plasma needs to be conducted within less than 4 h to prevent the release of genomic DNA due to cell lysis [8]. This is not always possible in oncological practices due to schedule conflicts or the lack of equipment such as high-speed centrifuges. Thus, blood samples need to be shipped to a testing laboratory, which requires specialized blood collection systems. Blood collection tubes containing a cell-stabilizing and nuclease inhibiting cocktail such as Cell-Free DNA BCT tubes (cfDNA BCTs) by Streck (La Vista, NE, USA) are suitable for shipping, as they are designed for up to 14 days whole blood storage at temperatures between 6 and 37 °C. The advantage of cfDNA BCTs over other cell stabilizing tubes on the market is the broad operating temperature range [8,9,10]. This enables an economical shipping box design, whereas users of blood collection tubes with temperature ranges between 18 and 25 °C need controlled room temperature (CRT) shipping solutions, e.g., phase exchange material, which increases shipping costs. Another advantage of the Cell-Free DNA BCT tubes (Streck tubes) is that they are known to be able to successfully maintain stable cfDNA levels at RT over time. Several studies have confirmed that RT storage prior to processing can be extended to 3 days [11], 4 days [9,10], 7 days [12,13,14,15], and even up to 14 days (in the case of fetal cfDNA levels) [16].

Aside from the wide application of cfDNA BCTs for noninvasive prenatal testing (NIPT) [17], only a few studies have reported on the utility of cfDNA BCTs for oncological applications. Promising results were already shown for colorectal cancer (CRC) [4], breast cancer [18,19,20], melanoma [21], and non-small-cell lung cancer (NSCLC) [22]. However, for all these studies, different pre-analytical conditions were chosen. The lack of harmonized pre-analytical standards [6,17,22,23,24,25,26] leads to deviating performances of cfDNA applications, which could be hindering reliable clinical data evaluation. Therefore, well defined and consolidated pre-analytical standards will ensure high-quality cfDNA samples for further downstream processes. Here, we propose a common consensus of pre-analytical steps using cfDNA BCTs for ctDNA analysis in different cancer types.

## 2. Materials and Methods

### 2.1. Ethics Statement

Samples for this study were either collected commercially from Indivumed GmbH (Hamburg, Germany), or in collaboration with University Hospital, Bonn. Indivumed GmbH obtained approval from the institutional review board of the Physicians Association of Hamburg, Germany (PV2963). The University of Bonn obtained approval from the Ethics Committee of the Faculty of Medicine of the University of Bonn. Written consent was obtained from all study subjects by the sample providers. All clinical data and samples were received by Sysmex Inostics GmbH anonymously.

### 2.2. Blood Collection

Venous blood from patients with CRC stage II–IV (*n* = 21) (study cohort I), advanced pancreatic cancer (*n* = 11) (study cohort II), and advanced stage NSCLC (*n* = 21) (study cohort III) was collected using standard phlebotomy techniques in both BD Vacutainer K_2_EDTA tubes (Becton Dickinson, Franklin Lakes, NJ; referred to as K_2_EDTA tubes) and Cell-Free I BCT tubes (Streck, La Vista, NJ, USA; referred to as cfDNA BCTs). Samples from cohort I were also used in a previous study [4], and were included in this study for a higher (*n*) as well as to show that the cfDNA BCTs showed comparable performance for various cancer types (i.e., CRC, NSCLC, and pancreatic cancer).

**Figure 1:**
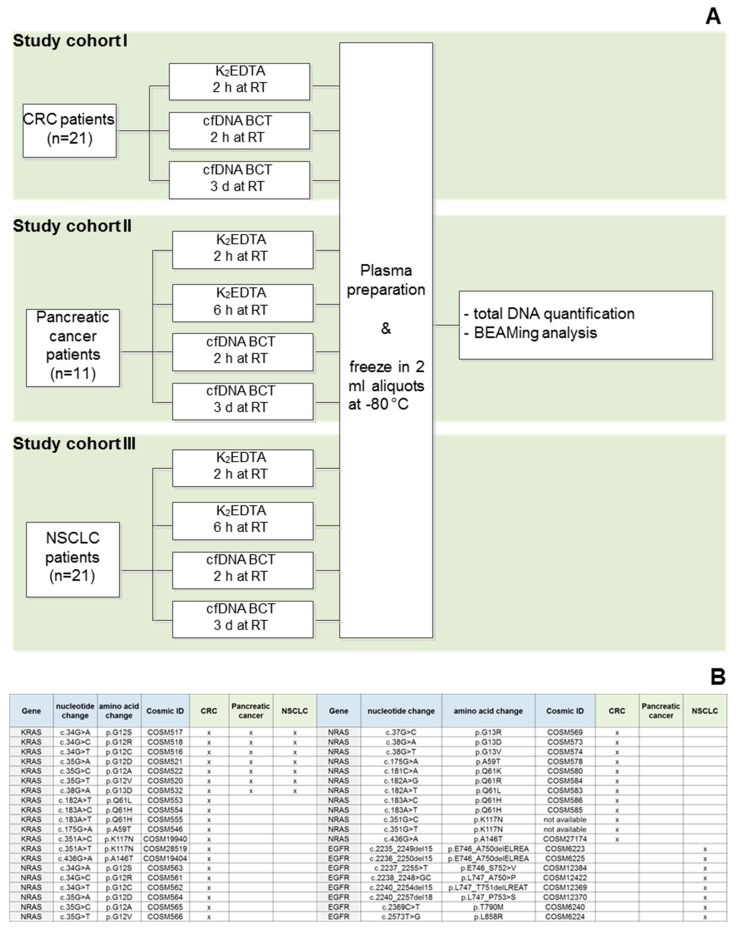
Experiment layout and study cohorts. (**A**) Experimental setup for evaluating the performance of cfDNA BCTs and K_2_EDTA tubes. Plasma samples from all patients were evaluated for cfDNA yield and mutational load using qPCR and BEAMing, respectively. Cohorts II and III included an additional storage condition (K_2_EDTA, 2 h at RT) to access nuclease activity with DNA quantification. (**B**) Analyzed mutations for CRC, pancreatic cancer, and NSCLC cohorts.

All blood collection tubes were filled to 10 mL as recommended by both manufacturers. Tubes were then inverted 10 times before room temperature (RT) transportation to the laboratory. Storage and processing conditions were performed according to the scheme in Figure 1.

### 2.3. Blood Storage

As shown in Figure 1A, blood tubes were stored for either 2 h (K_2_EDTA and cfDNA BCT) or 3 days (cfDNA BCT) before processing. In order to evaluate the nuclease activity, an additional K_2_EDTA tube was stored for 6 h before plasma preparation in study cohorts II and III.

### 2.4. Plasma Preparation

Following the indicated storage times, tubes were centrifuged at 1600× *g* for 10 min at RT using a swing-out rotor. To prevent contamination of the plasma with cells, a smooth braking profile was used, and the tubes were carefully removed. Without disrupting the buffy coat layer, the plasma fraction was transferred to a fresh 15 mL tube, leaving approximately 500 µL plasma in the blood collection tube. The supernatant was centrifuged a second time at 6000× *g* for 10 min at RT using a swing-out rotor and a smooth braking profile. The supernatant was again transferred to a fresh tube, leaving approximately 300 µL above the cell pellet, gently mixed by pipetting and aliquoted in 2.1 mL cryotubes. Plasma aliquots were frozen at −80 °C.

### 2.5. Cell-Free DNA Extraction

CfDNA from 2 mL plasma was extracted using the QIAamp Circulating Nucleic Acid Kit (Qiagen, Hilden, Germany). Differing from the manufacturer’s instructions, the proteinase K incubation time at 60 °C was extended from 30 to 60 min for both the cfDNA BCT and K_2_EDTA samples. This is recommended by Streck for plasma samples where blood was collected in cfDNA BCTs; to guarantee the comparability, this method was also applied to K_2_EDTA-collected samples. CfDNA was eluted in 140 µL AVE buffer and stored at 4 °C until qPCR and BEAMing analysis.

### 2.6. Cell-Free DNA Quantification

DNA was quantified using a qPCR assay specific for the LINE-1 sequence exactly as described in earlier publications [4,27]. Briefly, a short LINE-1 amplicon (96 bp) was used as a target for quantifying cfDNA fragments that exhibit a modal range of ~166 bp [28], while a larger LINE-1 amplicon (402 bp) was used as a target to quantify contaminating genomic DNA originating from white blood cells (WBCs) [29]. Because the short amplicon was selected from the sequence of the long amplicon, the ratio between the measured amounts of these amplicons (i.e., 402:96 bp ratio) served as a surrogate marker of genomic DNA contamination and cfDNA sample quality. Therefore, a high value indicates dilution of cfDNA with wild-type genomic DNA derived from peripheral blood cells lysed during storage or blood processing, while a low value indicates good cfDNA quality.

### 2.7. Mutation Analysis of cfDNA

CfDNA from CRC, pancreatic cancer, and NSCLC patients (*n* = 53), which was isolated from blood samples collected in both K_2_EDTA tubes and cfDNA BCTs, were analyzed for mutational load using BEAMing technology [30]. The BEAMing procedure was carried out as described previously [4]. As shown in Figure 1B, various common alterations in the KRAS, NRAS, and EGFR genes were tested for in the respective cohorts.

### 2.8. Statistical Analysis 

Statistical analysis using one-way ANOVA and linear regression with R2 calculation was performed in GraphPad Prism 6.07 (GraphPad Software, Inc., La Jolla, CA, USA). The *p*-values < 0.05 were considered to be statistically significant. Graphs were generated in GraphPad Prism.

## 3. Results

**Figure 2:**
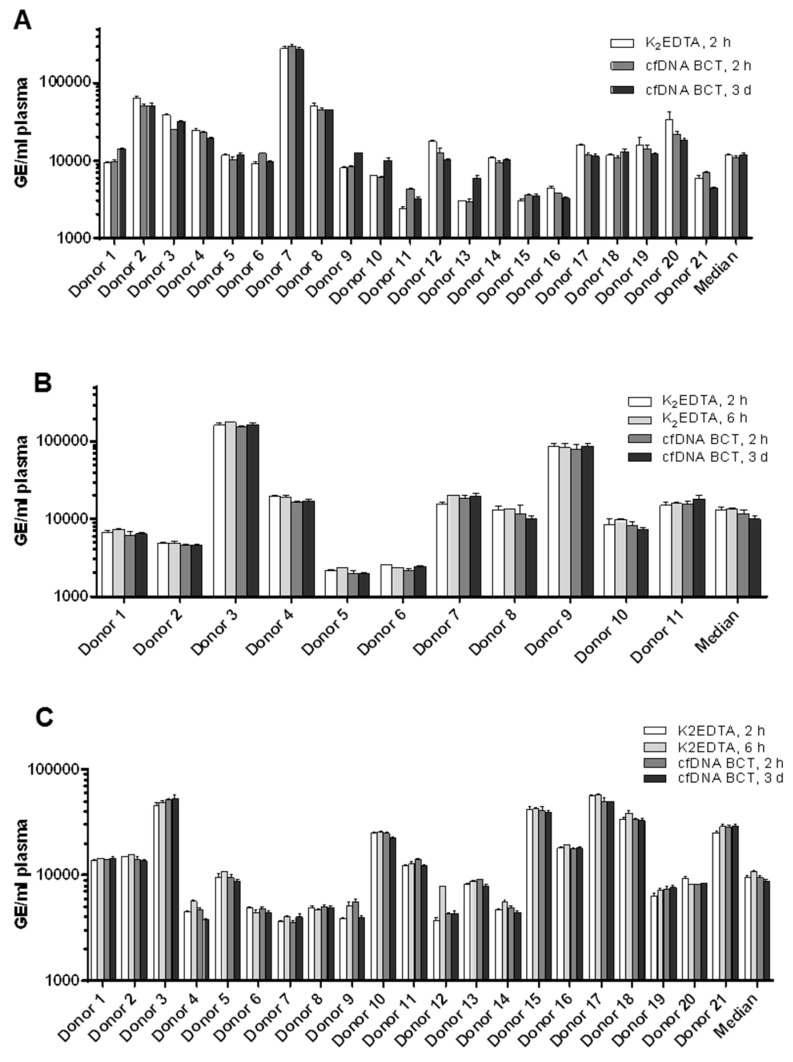
Evaluation of cfDNA yield. Plasma from CRC patients (**A**), pancreatic cancer patients (**B**), and NSCLC patients (**C**) was prepared after different whole-blood storage times at room temperature (18–22 °C) in K_2_EDTA tubes and cfDNA BCTs. CfDNA concentration in each plasma sample was determined with qPCR amplification using a 96 bp multi-copy LINE-1 fragment. Error bars indicate standard deviation.

As shown in Figure 1A, plasma was prepared after 2 h, 6 h, or 3 days storage at RT (18–22 °C) in K_2_EDTA tubes and cfDNA BCTs. For the analysis of cfDNA yield, a 96 bp multicopy LINE-1 fragment was amplified with qPCR. Figure 2 shows the cfDNA yields assessed for CRC patients (A), pancreatic cancer patients (B), and NSCLC patients (C). No significant differences were detected for cfDNA yields in the three study cohorts for samples collected in K_2_EDTA vs. cfDNA BCTs for all tested storage conditions. This shows that cfDNA BCTs were able to stabilize cfDNA in clinical samples for 3 days at RT. In addition, the pancreatic cancer samples, which were tested for higher nuclease activity, showed no significant change in yields between the samples stored for 2 and 6 h in K_2_EDTA tubes. Previous studies have also found that cfDNA BCT tubes were able to deliver stable yields of cfDNA after 2 [20], 4 [31,32], and 7 [33] days of storage at RT.

**Figure 3:**
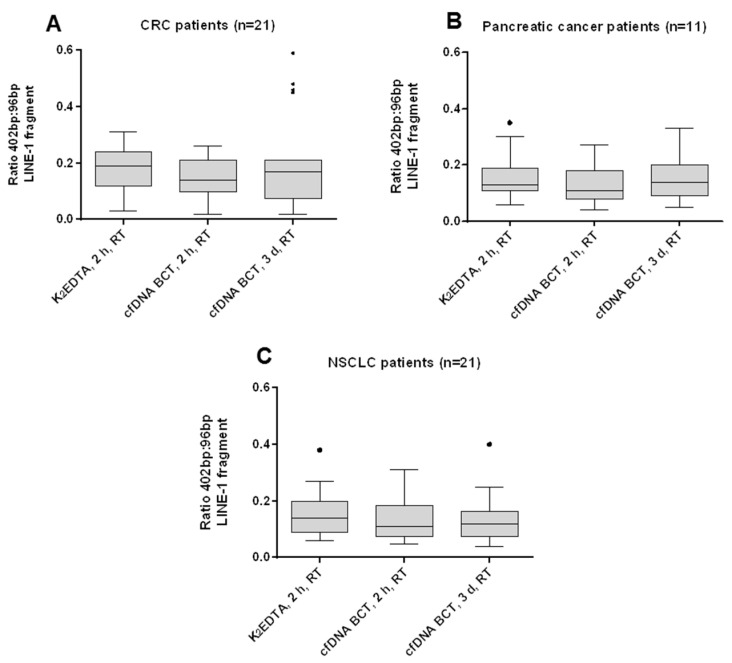
Effect of storage time on cfDNA sample quality. The level of genomic DNA contamination in plasma samples from (**A**) CRC patients, (**B**) pancreatic cancer patients, and (**C**) NSCLC patients following 2 h of RT blood storage in K_2_EDTA tubes, 2 h of RT storage in cfDNA BCTs, and 3 days of RT storage in cfDNA BCTs, was assessed by calculating the ratio between the measured amounts of long (402 bp) and short (96 bp) LINE-1 cfDNA fragments (y-axis). CfDNA BCTs were able to stabilize cfDNA and WBCs over 3 days of storage at RT. Statistical analysis was performed using Dunnett’s multiple comparisons test, and the Tukey method was used to create whiskers and outliers (black dots). No statistically significant differences were observed for all tested conditions (*p* > 0.05). Error bars indicate standard deviation.

To evaluate the effects of RT storage (18–22 °C) for up to 3 days in cfDNA BCTs, a ratio of a long (402 bp) and a short (96 bp) LINE-1 fragment was amplified and calculated as a gDNA/cfDNA ratio. According to Dunnett’s multiple comparisons test, no significant difference between long (402 bp) and short (96 bp) LINE-1 fragments was identified for blood stored for up to 3 days at RT in Streck cfDNA BCTs for (A) CRC patient samples, (B) pancreatic patient samples, or (C) NSCLC patient samples (*p* > 0.05) (Figure 3). We also did not identify significant differences between long and short LINE-1 fragments when blood was stored in BD K_2_EDTA for 2 h vs. 6 h for either pancreatic cancer or NSCLC samples (results not shown). Increased ratios compared with the K_2_EDTA reference values would have been indicative of a gDNA release or cfDNA decrease. Thus, qPCR results indicated cfDNA and WBC stability, thereby proving the ability of cfDNA BCTs to prevent gDNA contamination in clinical samples for up to 3 days’ storage at RT.

**Figure 4:**
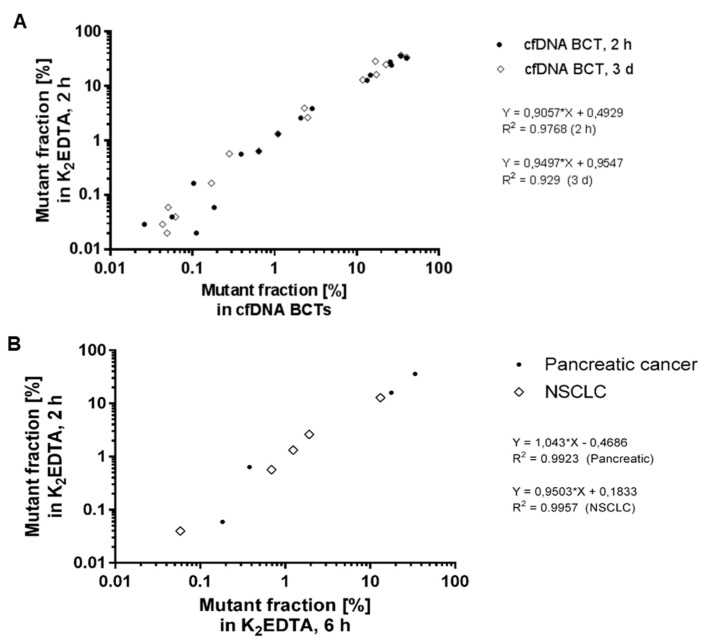
Correlation of BEAMing mutation test results between collection tubes. (**A**) Blood from CRC, pancreatic cancer, and NSCLC patients (*n* = 53) was collected in K_2_EDTA tubes and cfDNA BCTs, and samples were analyzed for mutational load using BEAMing after the indicated storage time at RT. Mutant fractions of all detectable mutation sets are plotted for both cfDNA BCT conditions (x-axis) and matched K_2_EDTA reference (y-axis). A total of 16 mutations were found in 15 of the 53 samples (28.3%), with one patient harboring a mutation in two genes. Mutant allele frequencies in cfDNA ranged from 0.026% to 41.6% and showed very comparable results between the tube conditions, even for low-positive samples. (**B**) Blood samples from NSCLC (*n* = 21) and pancreatic cancer patients (*n* = 11) were collected in K_2_EDTA tubes and processed at 2 h and 6 h following phlebotomy. BEAMing analysis revealed very comparable results, suggesting that K_2_EDTA tubes are sufficient for storage of samples for up to 6 h after collection. Good correlations were obtained for both pancreatic cancer and NSCLC samples, with R^2^ values of 0.9923 and 0.9957, respectively.

Blood samples from CRC, pancreatic cancer, and NSCLC patients (*n* = 53) were collected in K_2_EDTA tubes and cfDNA BCTs, and samples were analyzed for mutational load using BEAMing after preprocessing storage at RT (18–22 °C) for 2 h, 6 h, or 3 days (mutation analysis results are summarized in Table 1). Mutant fractions of all detectable mutation sets were plotted for cfDNA BCT conditions versus matched K_2_EDTA values as a reference. A total of 16 mutations were found in 15 of 53 samples (28.3%), with one patient harboring a mutation in two genes. Mutant allele frequencies in cfDNA ranged from 0.026% to 41.6% and showed very comparable results between the tube conditions even for low-positive samples (Figure 4A). Therefore, storage of clinical blood samples in cfDNA BCTs for up to 3 days did not impair the detectability of low-level mutations. These results were in accordance with previously published results, where cfDNA BCT tubes were found to successfully preserve samples for the detection of gene mutations in ctDNA in the plasma of CRC and NSCLC patients after storage at RT of 3 days [4,22] and up to 10 days for melanoma patients [21]. Additionally, blood samples collected from NSCLC (*n* = 21) and pancreatic cancer (*n* = 11) patients in K_2_EDTA tubes, and processed after 2 h and 6 h following phlebotomy, were analyzed for mutational load using BEAMing. Interestingly, samples stored for either 2 h or 6 h in K_2_EDTA tubes yielded comparable mutation testing results (Figure 4B). This suggests that the K_2_EDTA tubes are useful for mutation analysis of cfDNA from NSCLC and pancreatic cancer patient samples after up to 6 h of storage at RT (Figure 4B).

## 4. Discussion

Whether ctDNA is characterized for the purposes of basic research or clinical diagnostics, high-quality biospecimens are crucial for accurate and reliable measurements. One of the first steps that can be taken to ensure good sample quality is to select blood collection procedures that preserve the integrity of the ctDNA and prevent the disruption of peripheral blood cells, thereby limiting the release of wild-type gDNA and the subsequent dilution of the target molecules. Because the traditionally used K_2_EDTA tubes are not able to maintain the quality of ctDNA in blood samples when stored for extended periods prior to processing [10,14,16,19,22,31,32,34], we have in this study evaluated Streck cfDNA BCTs as an alternative method for blood storage. Proper storage conditions for longer time periods are crucial, because blood samples are often collected offsite and need to be shipped to the testing laboratory, which may be situated at a distant location.

In order to evaluate the efficacy of cfDNA BCTs in the context of clinical oncology, we characterized the cfDNA in plasma derived from blood collected from colorectal (*n* = 21), pancreatic (*n* = 11), and non-small-cell lung cancer patients (*n* = 21) in cfDNA BCTs. For each of the cohorts, blood was processed either immediately or 3 days following collection. Results from the cfDNA BCTs were compared with those from matching specimens collected in K_2_EDTA tubes. In addition, to test the effects of nuclease activity in a slight processing delay, we compared cfDNA measurements from blood samples collected from pancreatic cancer and NSCLC patients stored for 2 h vs. 6 h in K_2_EDTA tubes. CfDNA isolated from each of the above samples was characterized in terms of yield; level of gDNA contamination; and mutation status of KRAS, NRAS, and EGFR genes, as determined using BEAMing technology.

Results show that (i) cfDNA BCTs stabilize ctDNA for at least 3 days at RT in clinical samples (Figure 2), (ii) cfDNA BCTs prevent gDNA contamination for at least 3 days at RT in clinical samples (Figure 3), (iii) storage of blood in cfDNA BCTs for up to 3 days does not impair detectability of low-level mutations in any of the cancer patient cohorts (Figure 4A), and (iv) blood samples from pancreatic cancer and NSCLC patients stored for 2 h vs. 6 h in K_2_EDTA tubes yield comparable mutation testing results (Figure 4B). Taken together, the resulting data not only show the advantages of cfDNA BCTs over standard K_2_EDTA tubes pertaining to extended storage times, but also demonstrate the general suitability of cfDNA BCTs for specimen collection in clinical oncology. This is because cfDNA BCTs can be stored for extended time periods and can be shipped at room temperature, while at the same time conserving ctDNA sequence integrity and preventing gDNA contamination by stabilizing peripheral blood cells, which facilitates the detection of low-abundance mutations in cancer patients with various tumor types. These results are corroborated by several recent studies that have also demonstrated the utility of cfDNA BCTs [9,10,15,16,19,20,22,35]. However, some important caveats merit discussion here. First, as demonstrated previously, it is important that shipping temperatures do not fluctuate beyond the ranges recommended by the manufacturers, as only slight variations can result in a significant loss of peripheral blood cell stability and gDNA contamination [4,15]. As such, temperature-controlled shipping is required. Second, the diagnostic sensitivity and specificity of ctDNA have been shown to be increased by parallel assessment of other biomarkers and other genomic features of ctDNA [36,37,38]; however, cfDNA BCTs were not designed, optimized, and tested for the analysis of auxiliary biomarkers such as DNA methylation patterns [39], proteins, or cell-free RNA [40]. Last, in order to reverse the chemical fixations in these tubes, the DNA extraction procedure requires an extended heating step [10]. As such, ctDNA extraction methods that do not include or cannot accommodate an extended heating step may not be compatible with cfDNA BCTs. Despite these disadvantages, cfDNA BCTs significantly simplify and enhance the liquid-profiling-based mutational profiling of cancer patients, which has in the last decade proven extremely useful at various stages of the disease [41].

It is also important to note that cfDNA BCT tubes have been compared to other non-K_2_EDTA tubes, such as: (a) PAXgene tubes (PreAnalytiX GmbH, Hombrechtikon, Switzerland), which show similar efficacy for preserving cfDNA yield and integrity over extended storage times [32]; (b) Cell-free DNA collection tubes (Roche Diagnostics GmbH, Mannheim, Germany), which show performance similar to cfDNA BCTs in preserving cfDNA yield and integrity for up to 7 days of storage, after which samples stored in Roche cell-free DNA collection tubes demonstrated signs of gDNA contamination [35] (Another study showed that that PAXgene Blood ccfDNA Tubes (Qiagen), Cell-free DNA collection tubes (Roche), and cfDNA BCTs (Streck) performed similarly for the detection of EGFR T790M mutations with artificial spiked-in DNA fragments [42] after 7 days of storage.); (c) CellSave Preservative Tubes (Menarini Silicon Biosystems, Inc., Bryn Athyn, PA, USA), which were reported to perform similarly to cfDNA BCTs in their ability to stabilize both ctDNA and wild-type DNA after 48 [20] and 96 h [31] at RT.

## 5. Conclusions

The current pre-analytic study provides a firm basis for the reliable assessment of ctDNA yield as well as quantitative mutational analysis of the common genes KRAS, NRAS, and EGFR on plasma ctDNA even in very low concentration ranges. Stability in cfDNA BCT tubes was shown for at least 3 days and in conventional K_2_EDTA tubes for at least 6 h at RT, demonstrating excellent pre-analytical preconditions for future applications in routine oncology diagnostics.

## Figures and Tables

**Table 1:** Mutation analysis results from CRC, pancreatic cancer, and NSCLC samples collected in K_2_EDTA and cfDNA BCTs. Total DNA amount and mutant molecules refer to 2 mL plasma volume aliquots.

Cancer Type	Sample ID	Stage	Mutation	Mutant Fraction (%)	Total DNA Amount (GE)	Mutant Molecules
Colorectal	Donor 2, K_2_EDTA, 2h, RT	IVB	KRAS_g38a	32.83	117553	38593
	Donor 2, cfDNA BCT, 2h, RT			41.63	93975	39119
	Donor 2, cfDNA BCT, 3d, RT			40.84	94172	38457
	Donor 3, K_2_EDTA, 2h, RT	IVA	KRAS_g34t	27.86	70947	19768
	Donor 3, cfDNA BCT, 2h, RT			26.24	45306	11887
	Donor 3, cfDNA BCT, 3d, RT			16.87	58550	9877
	Donor 5, K_2_EDTA, 2h, RT	IIA	KRAS_g38a	0.020	21197	4,2
	Donor 5, cfDNA BCT, 2h, RT			0.112	18873	21
	Donor 5, cfDNA BCT, 3d, RT			0.049	21505	11
	Donor 7, K_2_EDTA, 2h, RT	IV	KRAS_g35a	24.36	518814	126362
	Donor 7, cfDNA BCT, 2h, RT			27.25	554853	151175
	Donor 7, cfDNA BCT, 3d, RT			22.70	501963	113966
	Donor 7, K_2_EDTA, 2h, RT	IV	NRAS_g34a	0.029	518814	151
	Donor 7, cfDNA BCT, 2h, RT			0.026	554853	144
	Donor 7, cfDNA BCT, 3d, RT			0.043	501963	216
	Donor 10, K_2_EDTA, 2h, RT	III B	KRAS_a183t	0.164	14514	24
	Donor 10, cfDNA BCT, 2h, RT			0.104	14883	15
	Donor 10, cfDNA BCT, 3d, RT			0.170	23001	39
	Donor 18, K_2_EDTA, 2h, RT	IVB	KRAS_g35t	3.883	28816	1119
	Donor 18, cfDNA BCT, 2h, RT			2.923	21746	636
	Donor 18, cfDNA BCT, 3d, RT			2.306	20766	479
Pancreatic	Donor 18, K_2_EDTA, 2h, RT	n/a	KRAS_1_g35t	0.632	11550	72.9
	Donor 18, K_2_EDTA, 6h, RT			0.381	13338	50.8
	Donor 18, cfDNA BCT, 2h, RT			0.647	10716	69.4
	Donor 18, cfDNA BCT, 3d, RT			0.641	11055	70.9
	Donor 06, K_2_EDTA, 2h, RT	n/a	KRAS_1_g35t	0.059	4629	2.7
	Donor 06, K_2_EDTA, 6h, RT			0.183	3725	6.8
	Donor 06, cfDNA BCT, 2h, RT			0.185	3269	6.1
	Donor 06, cfDNA BCT, 3d, RT			0.051	3952	2.0
	Donor 07, K_2_EDTA, 2h, RT	n/a	KRAS_1_g35a	35.99	28001	10078.7
	Donor 07, K_2_EDTA, 6h, RT			33.87	30269	10252.7
	Donor 07, cfDNA BCT, 2h, RT			35.32	33783	11930.7
	Donor 07, cfDNA BCT, 3d, RT			34.50	36337	12536.1
	Donor 09, K_2_EDTA, 2h, RT	n/a	KRAS_1_g35a	15.94	153645	24497.9
	Donor 09, K_2_EDTA, 6h, RT			17.82	145873	25994.9
	Donor 09, cfDNA BCT, 2h, RT			15.04	140710	21155.8
	Donor 09, cfDNA BCT, 3d, RT			17.33	157914	27367.4
NSCLC	Donor 03, K_2_EDTA, 2h, RT	n/a	KRAS_1_g35a	0.040	69619	27.6
	Donor 03, K_2_EDTA, 6h, RT			0.058	89446	52.2
	Donor 03, cfDNA BCT, 2h, RT			0.057	93901	53.1
	Donor 03, cfDNA BCT, 3d, RT			0.062	96664	59.8
	Donor 06, K_2_EDTA, 2h, RT	n/a	KRAS_1_g34t	0.564	8816	49.7
	Donor 06, K_2_EDTA, 6h, RT			0.690	7901	54.5
	Donor 06, cfDNA BCT, 2h, RT			0.396	8656	34.3
	Donor 06, cfDNA BCT, 3d, RT			0.280	7936	22.2
	Donor 08, K_2_EDTA, 2h, RT	n/a	KRAS_1_g35c	1.312	9025	118.4
	Donor 08, K_2_EDTA, 6h, RT			1.243	8654	107.6
	Donor 08, cfDNA BCT, 2h, RT			1.120	9162	102.6
	Donor 08, cfDNA BCT, 3d, RT			1.091	9005	98.2
	Donor 20, K_2_EDTA, 2h, RT	n/a	KRAS_1_g35t	12.71	15415	1958.9
	Donor 20, K_2_EDTA, 6h, RT			13.25	14294	1893.8
	Donor 20, cfDNA BCT, 2h, RT			13.70	14570	1996.6
	Donor 20, cfDNA BCT, 3d, RT			11.87	14914	1770.7
	Donor 21, K_2_EDTA, 2h, RT	n/a	EGFR_19_2236-50D	2.602	45839	1192.7
	Donor 21, K_2_EDTA, 6h, RT			1.921	52731	1013.0
	Donor 21, cfDNA BCT, 2h, RT			2.116	52190	1104.1
	Donor 21, cfDNA BCT, 3d, RT			2.533	53279	1349.3

## Data Availability

The data presented in this study are available in the article.
